# Asciminib in chronic myeloid leukemia: many questions still remain to be answered

**DOI:** 10.1038/s41408-021-00475-7

**Published:** 2021-04-29

**Authors:** Ahmet Emre Eşkazan

**Affiliations:** Division of Hematology, Department of Internal Medicine, Cerrahpaşa Faculty of Medicine, Istanbul University-Cerrahpaşa, Istanbul, Turkey

**Keywords:** Chronic myeloid leukaemia, Molecularly targeted therapy

Dear Editor,

Although a majority of patients with chronic myeloid leukemia (CML) in the chronic phase (CML- CP) achieve and maintain optimal responses under either imatinib and/or second-generation tyrosine kinase inhibitors (2GTKIs)^1^, some might seek alternative treatment modalities^2^. Ponatinib is a third-generation TKI, which can be utilized in this setting^3^, and in those harboring T315I mutation. In addition, allogeneic hematopoietic stem cell transplantation (allo-HSCT) is a reasonable and potentially curative option, which can be chosen in suitable cases^4^.

Asciminib, the first-in-class STAMP (**S**pecifically **T**argeting the **A**BL **M**yristoyl **P**ocket) inhibitor, may serve as an efficacious and relatively safe option in CML patients with resistance to the previous TKIs^5,6^. And recently, the efficacy and safety results of the multi-center, phase III ASCEMBL trial, comparing asciminib vs. bosutinib in patients with CML-CP receiving ≥2 lines of previously TKI treatment were shared^7^. In this regard, Garcia-Gutiérrez and colleagues^8^ published a multi-center study reporting the first “real-life” data on the use of asciminib in 31 CML patients, who experienced treatment failure (both resistance and intolerance) with multiple lines of TKI therapy.

As this is a very informative study with very interesting findings, there are still some points that need to be further underlined.

- Most of the patients (*n* = 22, 71%) in this cohort were switched to asciminib due to intolerance, leaving nine patients (29%) in the TKI-resistant group^8^. In addition, it was stated that eleven patients (35%) had received ponatinib prior to asciminib. Three patients with prior ponatinib use achieved major molecular response (MMR) or deeper responses under asciminib, of whom two were in the TKI-intolerant group and one in the TKI-resistant group. So, I guess the remaining eight patients with previous ponatinib use were in the TKI-resistant group, and as a result, all patients in this group had received ponatinib^8^. Taken all these together, only two patients in the TKI-intolerant group had previous ponatinib exposure and although the inclusion criteria were listed in the paper^8^, since they were not specifically mentioned, what were the reasons to choose asciminib but not ponatinib, in the remaining 19 patients in the TKI-intolerant group – comorbidities, previous TKI-related toxicities, etc.?
- Similarly, although it was recently shown that asciminib can be a reasonable option in patients harboring T315I^9^, in their cohort, Garcia-Gutiérrez and coworkers^8^ reported that there was one patient with a baseline T315I mutation, who lost hematologic response under asciminib therapy. I was wondering whether this patient had received ponatinib previously or not. If not, why was asciminib chosen over ponatinib?
- The authors stated that two patients experienced blast crisis under asciminib, and two patients died due to disease progression^8^. Were they the same two patients and how were the cases with blast crisis managed? This information is important because we also had a patient in the ASCEMBL trial^7^, who experienced a myeloid blast crisis under asciminib therapy. Our patient received ponatinib after asciminib failure, which induced complete cytogenetic response (CCyR), and then he underwent allo-HSCT in the second CP.
- In the study of Garcia-Gutiérrez and colleagues^8^, cumulative CCyR and MMR rates were 48 and 33%, respectively. These response rates were superior in the TKI- intolerant group than those observed in the TKI-resistant group. And after a median follow-up of 10.2 months, 27 patients (87%) remained on asciminib treatment. Knowing the fact that allo-HSCT can be administered in these patients^2^, do the authors consider asciminib as a bridge to allo-HSCT in transplant-eligible patients since no data on allo-HSCT was shared in their paper? Or do they prefer to continue asciminib in patients who achieve and maintain optimal responses regardless of transplant eligibility?

Asciminib is a potential treatment approach as stated in the most recent European LeukemiaNet recommendations^3,10^. It previously showed promising results in heavily pretreated patients with acceptable toxicity profile^6,7^, and in line with these findings, Garcia-Gutiérrez et al.^8^. demonstrated that asciminib therapy was also efficient and safe in CML patients with multi-TKI failure in daily clinical practice. Patients who switched to asciminib due to intolerance had superior response rates than cases who experienced prior TKI resistance, which was compatible with previous TKI studies^11^.

Although asciminib can be beneficial in CML cases who are resistant to multiple TKIs, for my point of view, it can be particularly used in patients with prior TKI failure including ponatinib, especially if the TKI switch is due to intolerance. As ponatinib is recommended in patients who are resistant to at least one 2GTKI^3^, on the other hand, in a ponatinib-naive patient, following a 2GTKI failure, asciminib can also serve as an alternative treatment option, especially in those with cardiovascular risk factors that prevent ponatinib use^3^.

In addition, for those harboring T315I mutation, the preliminary results of a phase I study showed that asciminib monotherapy at the 200 mg BID dose induced encouraging results with generally manageable toxicity^9^. MMR attainment was higher in ponatinib-naive patients compared to ponatinib-pretreated ones, both by 24 weeks (57.1% vs. 28.6%) and at 60 weeks (estimated: 66% vs. 32%).

Notwithstanding the superior efficacy of asciminib over bosutinib in patients who experienced treatment failure to at least one 2GTKI^7^, maybe in the real-life setting, the more practical “competitor” of asciminib could be ponatinib, and future studies comparing asciminib with ponatinib in patients with 2GTKI failure are definitely warranted. As stated by the authors, there are still many questions that remain to be answered regarding the use of this promising drug among CML patients.
